# Canine perspective taking: Anticipating the behavior of an unseen human

**DOI:** 10.1016/j.isci.2025.111811

**Published:** 2025-01-16

**Authors:** Ludwig Huber, Pauline van der Wolf, Machteld Menkveld, Stefanie Riemer, Christoph J. Völter

**Affiliations:** 1 Comparative Cognition, Messerli Research Institute, University of Veterinary Medicine Vienna, Medical University of Vienna and University of Vienna, 1210 Vienna, Austria; 2 Department of Comparative Cultural Psychology, Max Planck Institute for Evolutionary Anthropology, 04103 Leipzig, Germany

**Keywords:** Canine behavior, Biological sciences, Behavioral neuroscience

## Abstract

Although dogs exhibit remarkable capabilities for interacting with humans, the underlying cognitive mechanisms remain insufficiently understood. Here, we investigated canine perspective taking by challenging dogs to decide whether and where to steal food in the absence of a human who had prohibited them from doing so. The dogs could only infer the experimenter’s presence through a sound (carrot chopping) they had perceived during a prior exploration phase, in which they also had the opportunity to observe from which locations in the room the human was visible. In the test, the majority of dogs preferred to steal from a plate that was not visible from the location where the human had chopped carrots before when they heard a playback of the chopping sound but not when they heard a control sound (street noise). These findings provide evidence that dogs anticipate the behavior of humans without relying on observable visual cues.

## Highlights

• In a stealing task, dogs had to choose between two plates with food
• During prior exploration, only one of the plates was in line of sight of a human
• In the test, the human was absent but indirect auditory cues signaled her presence
• Despite being alone, dogs stole forbidden food from where they could not be seen

## Graphical abstract

**Figure:**
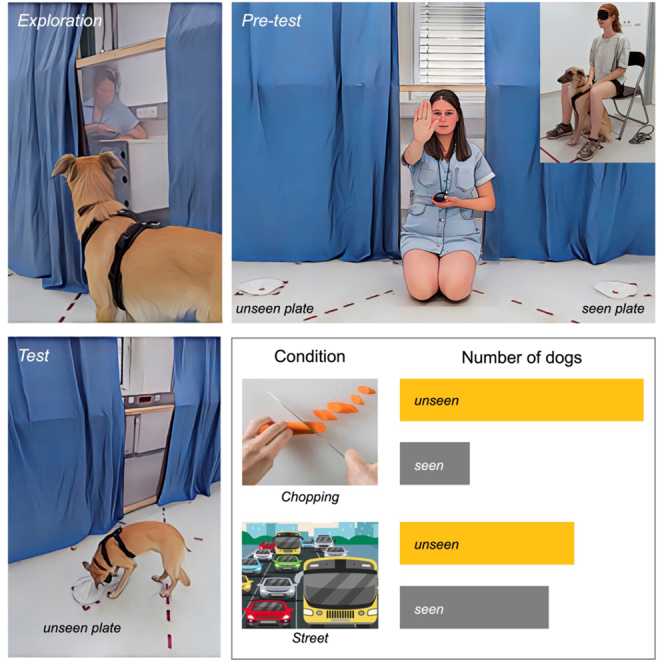

## Introduction

How do dogs comprehend human behavior? Answering this question holds significance not only for comparative psychologists, evolutionary biologists, and neuroscientists but also for individuals involved in the training, breeding, and companionship of dogs. Empirical evidence has increasingly indicated that dogs have developed, through both domestication and cohabitation with humans, a remarkable sensitivity to our behaviors.^1^ This sensitivity can be attributed to two primary factors: first, through human selection over millennia, dogs have honed their ability to interpret human gestures, speech, and behavior^2^; second, through learning, they accumulate extensive experiences throughout their lives alongside humans, characterized by close, intimate relationships.^3^

For instance, dogs demonstrate the ability to visually discern and recognize human faces,^4^ as well as to identify and interpret facial expressions.^5^ They also gather significant social information through various communicative gestures, such as pointing,^6^^,^^7^ in addition to assessing human attentiveness.^8^^,^^9^^,^^10^^,^^11^

Dogs use a multitude of cues to interpret human attentional states.^12^ Among these cues are the human’s body orientation, head direction, and, crucially, gaze direction, all of which are fundamental indicators of human attention in communicative contexts.^13^^,^^14^ Gaze direction not only aids dogs in understanding the general attentional state of the human (e.g., alertness and vigilance) but also serves a referential function, indicating which elements of the environment capture human interest.^15^ This second level of gaze responsiveness involves following another’s gaze toward distant objects^16^—a skill that has been demonstrated in dogs during object-choice tasks.^17^ A preference for focusing on object-directed gaze, as opposed to gaze directed into open space, suggests that dogs possess an understanding of the referential nature of looking, akin to that observed in human infants.^18^

Even more sophisticated is geometrical gaze-following, which entails tracking the gaze direction of others around visual obstacles.^19^ Dogs are among the few nonhuman species, besides a few select non-primate species and certain corvids,^20^ that appear to possess this aptitude.^21^ It has been argued that this ability stems from an innate capability that has been selectively enhanced through the process of domestication.^22^ Collectively, there is substantial evidence underscoring that dogs are sensitive to behavioral and environmental cues associated with human visibility and attentiveness (for a review, see Huber et al.^23^). Dogs not only recognize and appropriately respond to a variety of visual attention cues but also demonstrate a burgeoning understanding of the human visual perspective^24^ and exploit this information in a functional way both in cooperative, e.g., begging,^12^ and competitive, e.g., stealing,^8^^,^^9^^,^^25^ contexts.

Tomasello and colleagues^26^ posited that the active utilization of gaze cues from others is a pivotal advancement in the understanding of mental states, such as attention and intention. A further progression toward mentalizing, often referred to as “theory of mind,”^27^ necessitates inferential abilities based solely on indirect information. Notably, research exploring the capacity to transcend mere associations between specific behavioral or environmental cues and potential outcomes remains sparse. In the context of Guesser-Knower studies, researchers have presented subjects with tasks where behavioral cues vary across different conditions (e.g., when the guesser is absent or with eyes covered or the head turned away while the knower is present or with the eyes uncovered or the head oriented toward the hider, respectively).^28^^,^^29^

In a unique investigation, both informants exhibited identical behaviors, gazing in the same direction toward a specific location in the room.^30^ However, the informants varied in their local positioning, particularly in relation to the hider’s location—which provides environmental cues. Dogs preferentially followed the pointing cue provided by the knower, consistent with the notion that they employed geometrical gaze-following to discern which of the two informants had observed the concealment of food. This performance might be regarded as the ability to infer the differential perceptual access of others in the absence of gaze cues, thus a kind of perspective-taking ability. Nonetheless, the dogs’ choice behavior could have been based solely on responding to “observables”—stimuli that were physically present—rather than about mental states.^31^ According to Heyes,^32^ dogs could, but they need not, “understand” or have a “theory” about why the relationship between the position in the room and the knowledge of where food is hidden holds; they need not explain it to themselves with reference to what the “knower” has “seen” and therefore “knows.” But if we exclude “mindreading,” is the only alternative “behavior reading”? Perhaps not. Consequently, to overcome the “observables” problem by attributing visual access to others without relying on observable visual cues, it becomes essential to eliminate or control for all visually accessible differences that the perceiver could leverage to identify the knower.^32^^,^^33^^,^^34^^,^^35^

As a first attempt, researchers have tried to develop designs that control for others’ gaze.^36^ In an elegant study with common ravens, Bugnyar et al.^37^ demonstrated that these birds assessed the visual access of conspecific competitors in scenarios where they themselves could not see them. By extrapolating from their personal perceptual experiences and discerning the sounds of fellow ravens, these birds inferred the possible presence of a competitor and the likelihood of being watched. This inference was based on the presence of an open peephole in the wall separating their compartments. The authors concluded that ravens possess cognitive capabilities that extend beyond mere behavioral interpretation.

In the realm of food caching, corvids exhibit heightened sensitivity to the presence of conspecifics who may pilfer their caches, suggesting that their perspective-taking abilities have been under significant selective pressure throughout evolution. This raises an intriguing question: can other nonhuman species that did not evolve under these specific selective pressures also attribute visual access without relying on visual behavioral cues?

Accurately assessing the visual access of others to a target object offers significant advantages. Such discernment can inform a dog’s choice regarding whom to trust as an informant in cooperative tasks or, alternatively, to strategize counter-tactics for securing a desirable object in competitive scenarios. Indeed, dogs have successfully navigated a multitude of such tasks with speed and reliability across diverse conditions, demonstrating capabilities such as geometrical gaze-following,^21^ stealing in the dark,^25^ concealing information from others,^38^ and guesser/knower differentiation.^29^^,^^30^

Recently, dogs were able to differentiate between misleading human informants possessing either true or false beliefs.^39^ But still, do these examples justify the conclusion that the subjects who solve the false belief test know about the mental states that mediate behavior or merely know about behavior^40^? For instance, they might have learned that people search for an object where they last saw it and not necessarily where the object actually is. Such a behavioral rule, whether learned or innately predisposed for, would explain the subject’s understanding of the link between the behavior of not having seen the object in its new location and the likely action of looking in the wrong place.^41^ In sum, there seems to be a continuum between behavior reading and mind reading, between responding to directly observable features of the other and understanding the other’s intentions, believes, and desires based on a full-blown theory of mind. A step away from merely reacting to behavioral cues from any perceptual modality—visual, olfactory, auditory, haptic, or any other—toward perspective taking would be the ability to combine innate or learned behavioral rules flexibly with newly acquired information, even if this information is perceived in a different perceptual modality.

**Figure 1:**
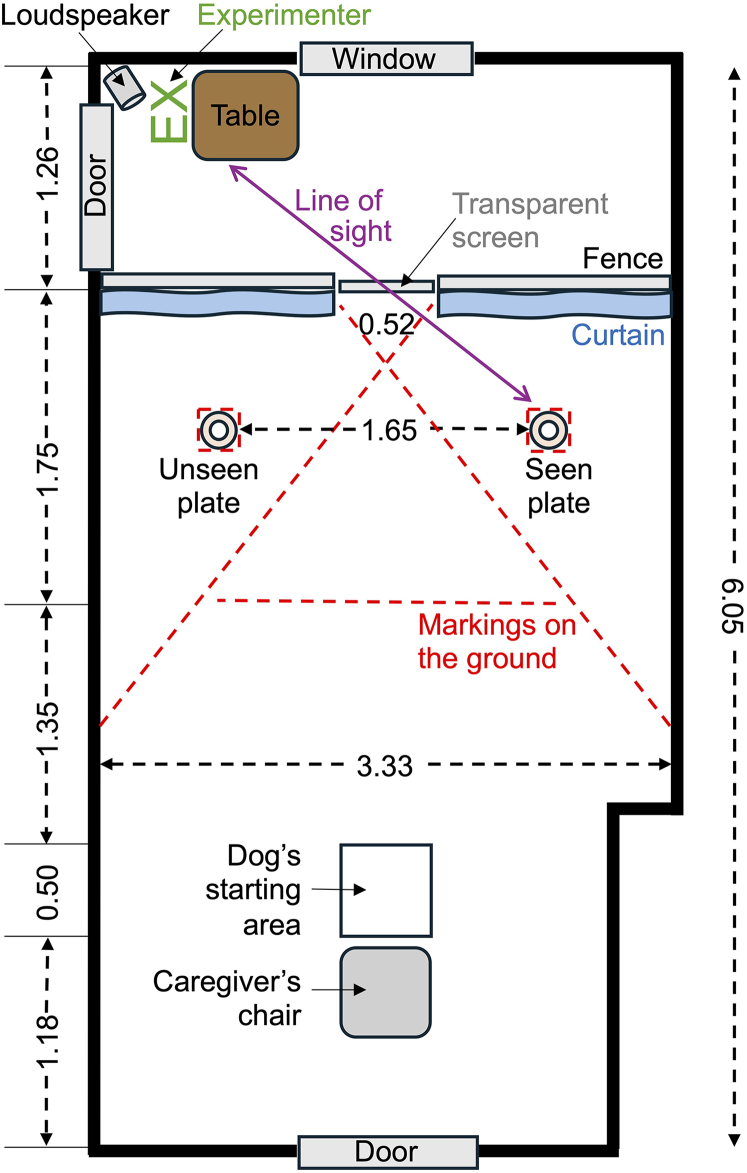
Scale drawing of the test room in the Clever Dog Lab Vienna with corresponding legend and measurements, showing the curtains and fence with the transparent screen that divide the room in the dog’s testing area (with the starting position of the dog) and the experimenter’s “kitchen” area (the position of the experimenter at the table), the position of the loudspeaker, the two food plates, the line of sight, and markings on the ground

**Figure 2:**
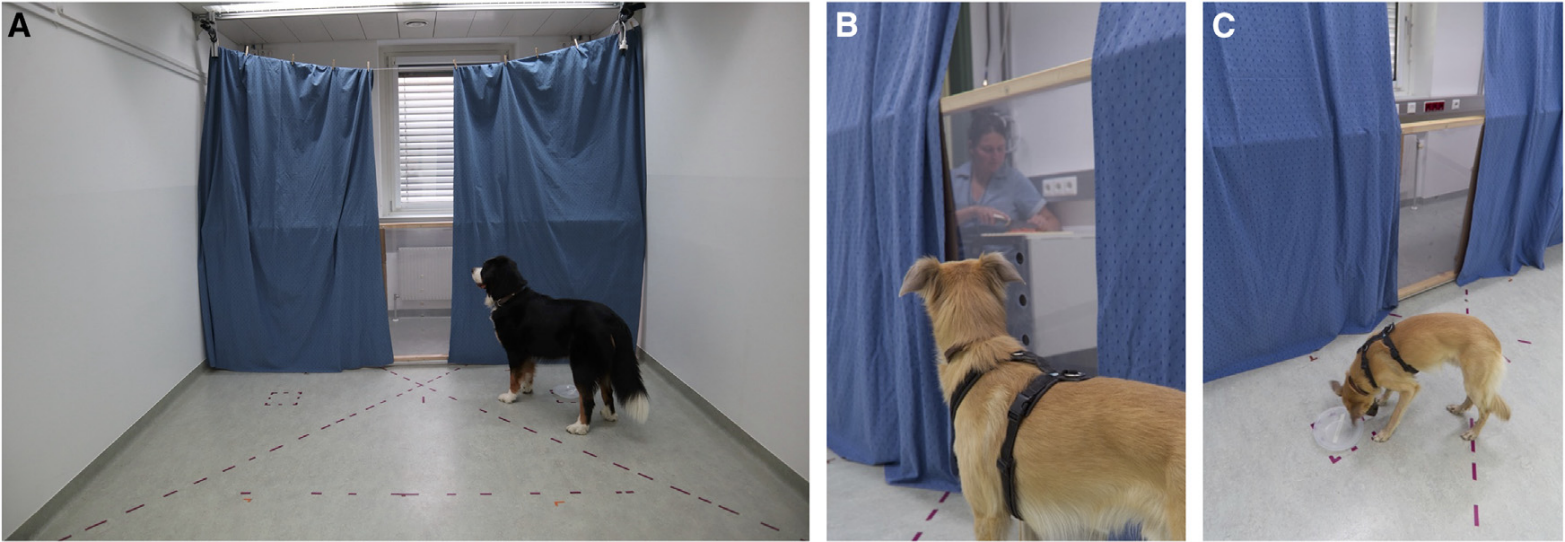
Still photos from the exploration phase in a trial with the food plate on the right wall from the dog’s perspective (location A) (A and B) Dogs can see through the gap between the curtains the human chopping carrots. (C) The dog is feeding from the plate at location B where it cannot see the human.

**Figure 3:**
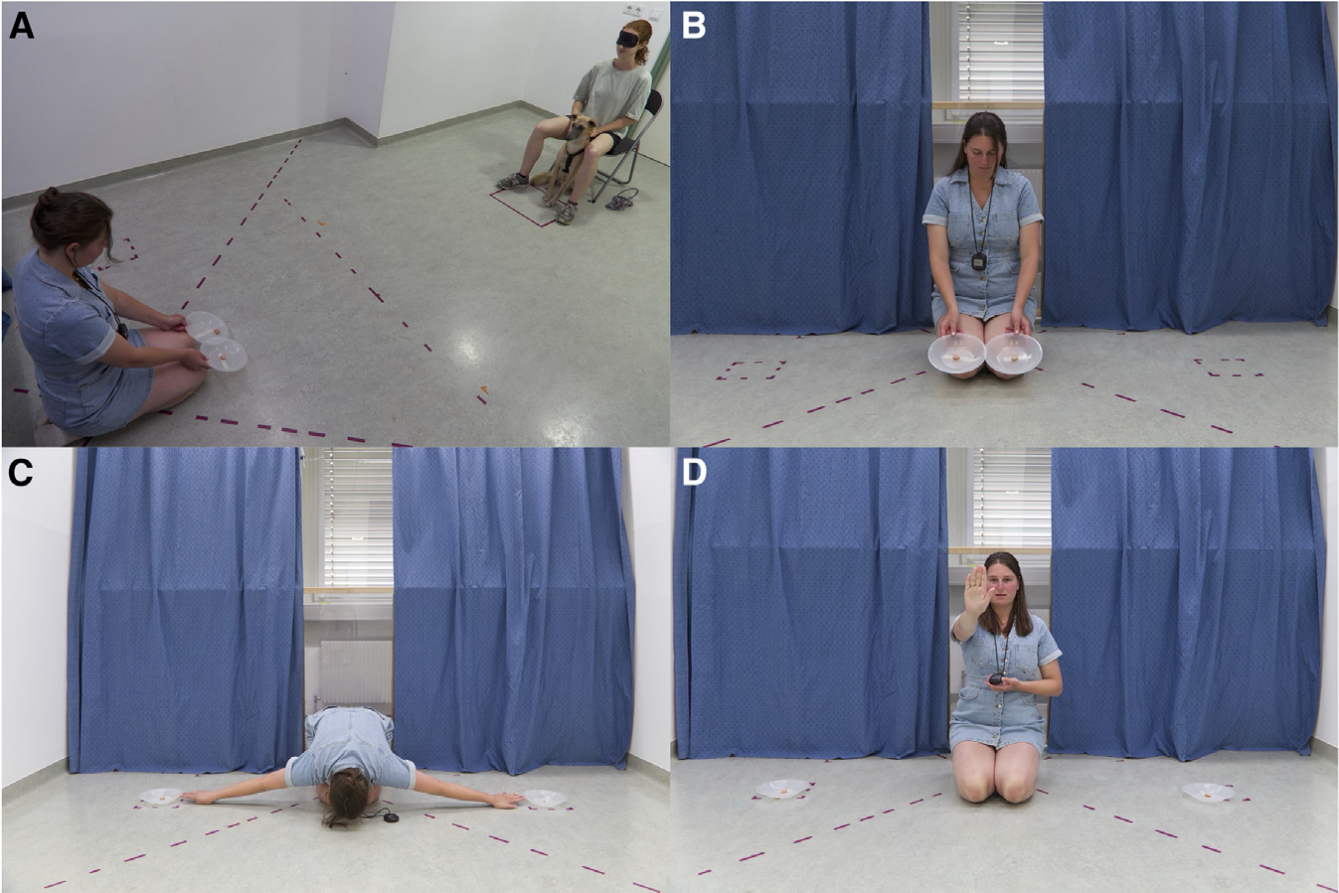
Still photos from the pre-tests (A–D) The experimenter shows the two plates baited with one piece of sausage each (A and B) and then moves them to the final position (C) and finally tells the dog (accompanied by a hand gesture) not to take the food (D).

Here, we tested whether dogs take into account the visual access of humans, even when they cannot see them, modeled after the design employed in the raven study.^37^ Instead of evaluating cache protection strategies, dogs were presented with a scenario where they could steal food that they had previously been forbidden to take by a human experimenter at two spatially separated locations (Figure 1). When deciding between the two locations, dogs were unable to rely on (visually) observable features, such as open eyes, gaze orientation, or line of sight, because the human experimenter was absent in the test. However, they could hear a “carrot chopping” sound that had been paired in a single exploration trial with the presence of the human experimenter (Figure 2). So, if the dogs remembered from the pretest that they are not allowed to take the food (Figure 3), then inferred the human presence based on the chopping sound and also could transfer their prior visual experience of the location in the room where they had observed the human chopping carrots to the test situation, we expected them to choose the out-of-sight food source in this test condition (“hidden stealing hypothesis”).

As a control, we tested a second group of dogs exposed to street sounds, a sound cue that should not elicit the expectation that the human was nearby. Thereby we intended to create a condition in which the dogs would likely feel secure and, consequently, would exhibit no particular preference in choosing between the food sources.

## Results

### Choice of (first approached) plate

**Figure 4:**
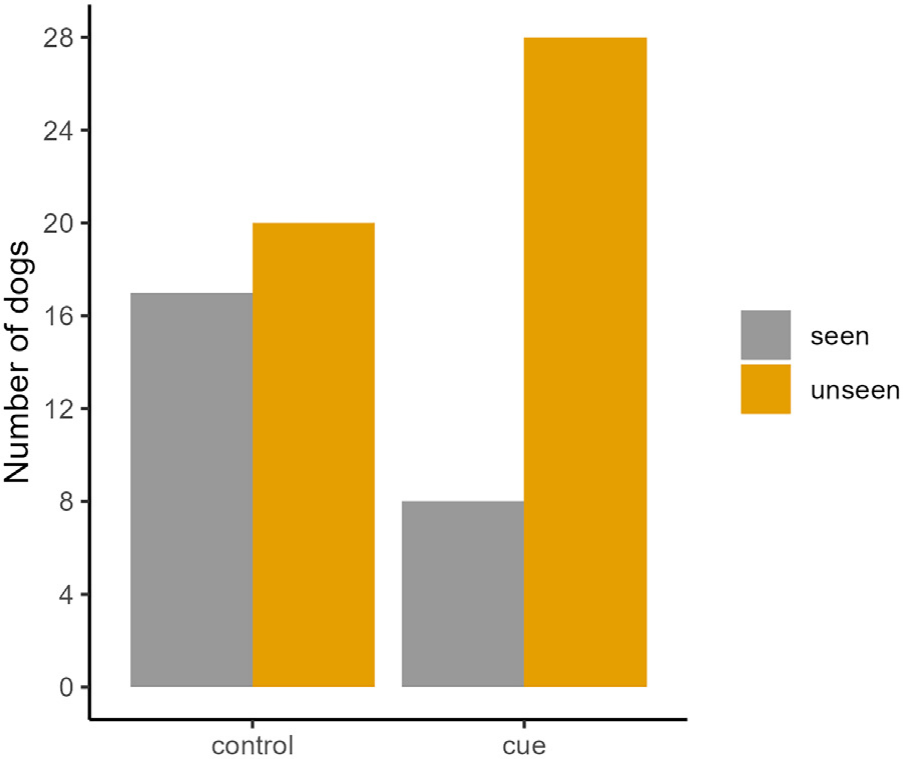
Bar plot showing the number of dogs by condition that approached the seen and unseen plate first in the test phase (*n* per condition: control condition: 37 dogs; cue condition: 36 dogs)

In line with our predictions, we found that significantly more dogs (78%) chose the unseen plate first in the cue condition than in the control condition (54%; χ2(1) = 4.85, *p* = 0.028; Figure 4 and Table S1). The control predictors age (χ2(1) = 0.26, *p* = 0.608) or sex (χ2(1) = 0.79, *p* = 0.375) had no significant effect on the dogs’ choice performance.

Dogs in the cue condition were significantly more likely to choose the unseen plate first (28 out of 36 dogs, binomial test: *p* = 0.001), unlike the dogs in the control condition whose performance did not deviate significantly from the chance level of 0.5 (20 out of 37 dogs, *p* = 0.743).

### Latency to approach the first plate

When analyzing the dogs’ latency to approach the first plate, we found no significant interaction between condition and the plate the dogs had chosen (scaled deviation (dev.) = 0.01, degree of freedom [df] = 1, *p* = 0.942), nor main effects of condition (scaled dev. = 0.02, df = 1, *p* = 0.890; Table S2), the chosen plate (scaled dev. = 0.50, df = 1, *p* = 0.477), age (scaled dev. = 0.72, df = 1, *p* = 0.396), or sex (scaled dev. = 0.01, df = 1, *p* = 0.908).

## Discussion

The results of the stealing test align with our hidden stealing hypothesis: 28 out of 36 dogs exposed to the chopping sound targeted the unseen food plate first in an attempt to steal the high-value food. Dogs that were exposed to ambient street noise did not show a similar preference for the unseen plate. This suggests that the dogs in the chopping condition expected the experimenter’s presence on the basis of the specific auditory cue of carrot chopping. The sound of chopping was associated with the experimenter because the dog has seen and at the same time heard the experimenter chopping in the exploration phase when visiting the seen plate. Alternatively, the dogs might have built an association between this sound and the presence of humans in their previous life. In any case, this memory seemed to have controlled the dogs’ behavior in the test (sound-cue) condition. Upon hearing the chopping, they chose the unseen plate significantly more often than the seen plate, even though this plate was physically closer to the chopping noise associated with the person. Thus, the dogs were not just avoiding the experimenter they heard but seemed to attempt to remain out of her sight—or to avoid seeing her—when taking the food.

But how did the dogs make their decision? Did they infer from their previous experience in the exploration phase that the experimenter, when sitting at the table chopping carrots, could see the “seen” plate and would thus be likely to interfere and remove the plate like in the first and third pre-test (see STAR Methods section)? In other words, did the dogs attribute visual access to the experimenter? Or did they (just) remember from where in the room they themselves could see the human chopping carrots? While the first explanation is advocating a psychologically rich, allocentric perspective-taking solution (“you can see me at the location where I have seen you previously”), the second one might be psychologically leaner and egocentric, an outcome of previous associative learning.^42^^,^^43^ For instance, dogs might have learned the heuristic that humans respond to their behavior when they are in line of sight but not otherwise (“you will respond at the location where I can see you”). Therefore, they might avoid sight lines associated with the researcher being visible.

A previous study indicated that dogs stole more food when they were hidden from a human’s view by a large barrier compared to a small one or when the barrier featured a small window.^44^ This led the authors conclude that dogs use an allocentric perspective rather than egocentric perspective to gauge the visual access of others under various conditions. Similarly, dogs refrained from stealing forbidden food that was illuminated (together with the surrounding area) as opposed to in the dark, suggesting that they recognized the potential visibility of their actions to the human.^25^ This was again interpreted by the authors as the possibility that dogs take into account the human’s visual access to the food while making their decision to steal it. And also in a fetching task, in which dogs developed a preference for the one of two toys that was visible to the human, although both were visible for the dog, they appeared to be sensitive to others’ visual access, even if that differed from their own.^45^ Later, however, these studies have been re-interpreted by advocating egocentric strategies as the more likely ones to explain the previous studies.^22^

Although in the current study the egocentric explanation of the dog’s behavior may be considered more parsimonious as well—it does not require attributing visual access to others—it still requires that the dogs remembered from the single trial in the exploration phase that they could see the human experimenter (chopping carrots) through the gap in the curtain when eating dry food from the right but not the left plate. This is not something they could have learned through trial and error, but they had to rely on this one-event memory to know at the moment of choice that they would be in the human’s line of sight near the right plate, thus potentially risking a response by the human experimenter. Nonetheless, when making their choice (whether and where to go), they were not in the line of sight of the expected human experimenter but could only rely on their experience from the exploration phase. Consequently, they could not directly perceive (“read”) the behavior of the (actually absent) human experimenter at the moment of choice.

The subjects in our cue condition used cues from the auditory modality (the chopping sound) and the memory of a single, short-term visual perception (seeing the experimenter from a specific location) to make a delicate decision (if and where to steal food) in a novel situation (a lab) with a non-visible (actually absent) stranger (the experimenter). Thus, like ravens,^37^ the dogs seemed to combine these multi-modal (visual and auditory) cues to quickly determine the safe “unseen” option. Still, we cannot entirely exclude the possibility that prior experience with similar stimuli (e.g., chopping sounds) or spatial arrangements allowed them to adapt to this situation so swiftly.

It is noteworthy that the data do not allow to discern between the egocentric interpretation (following a previously learned behavioral rule) and the allocentric interpretation (taking the perspective of the experimenter, based on the memory of what they had seen at the “seen” plate). Admittedly, complex behavioral outcomes are possible when egocentric heuristics and memory processes are combined with exposure to complex but reliable environment-behavior relationships. Unfortunately, recent studies on perspective taking in dogs do not provide a clear picture but point in one direction or the other depending on the task at hand. On the one hand, the findings of dogs discriminating between guesser and knower^29^ suggest a basic perspective-taking ability. This was confirmed by dogs that identified the knower although both informants were looking in the exact same way.^30^ And the strongest evidence for perspective taking so far was found in a study in which dogs behaved differently in response to the same misleading suggestion from human informants with either a true or false “belief” about the location of hidden food, who differed solely in the timing of leaving and re-entering the room.^39^ On the other hand, dogs did not show perspective-taking abilities in a study in which the only available cue to infer others’ visual access consisted of the subjects’ own previous experience with novel visual barriers.^46^

As pointed out by Tomasello and Call,^47^ some theorists prefer leaner interpretations (so-called scoffers) while others prefer richer interpretations (so-called boosters) of the behavioral/cognitive processes in nonhuman animals. The scoffers think that nonhuman animals respond to the behavior of others but do not conceive of any cognitive processes underlying this behavior. Boosters, in contrast, argue that there is rarely any concrete evidence that animals have had the requisite experiences to learn the required behavioral rules—there is just a theoretical possibility. They propose that it is more plausible to hypothesize that some nonhuman animals really do know what others do and do not see in many circumstances. It seems that a few more cleverly designed, well-controlled, statistically robust, and easily replicable studies are needed to determine which cognitive tools dogs actually use to understand their human counterparts.

### Limitations of the study

An alternative, lower-level explanation for the dogs’ preference for the unseen plate during the sound-cue condition is that they favored approaching the side from which they could hear the chopping sound due to a previously formed association between the sound and food. However, it is improbable that many dogs would connect the sound of chopping with the preparation of dog food, as most dogs are either fed commercial dog food or items that require minimal preparation. The association with chopping would only be formed if feeding the dogs is temporarily connected with chopping significantly more often than chopping with anything else.^48^ And it cannot explain why the dogs would not approach the window where they could actually see the source of the chopping if they were only attracted by the chopping sound. Therefore, we consider this alternative explanation to be less likely than the “hidden stealing” hypothesis. But to be on the safe side it would be necessary to control for the low-level possibility by using a sound produced by the experimenter uniquely in the exploration phase, with which dogs could not have formed associations in their previous life, or by testing whether naive dogs would prefer a plate located near the source of a chopping sound over a plate further away. In any case, there are numerous other ways to test the ability of dogs to infer (1) the presence of a human without direct visual information and (2) what the human can or cannot see based on previous encounters with her and the prevailing spatial conditions.

## Resource availability

### Lead contact

Further information and requests for resources should be directed to and will be fulfilled by the lead contact, Ludwig Huber (ludwig.huber@vetmeduni.ac.at).

### Materials availability

Detailed description of the materials is listed in the STAR Methods section. Any further information is available from the lead contact upon request.

### Data and code availability

• All data, code, and materials used in the analyses are available on a public repository (DOI: https://doi.org/10.5281/zenodo.13797455).
• Additional information: any additional information required to reanalyze the data reported in this paper is available from the lead contact upon request.

## Acknowledgments

The authors would like to thank the Clever Dog Lab manager, Karin Bayer, for her help at the lab; Markus Fitzka for helping to build the setup; and Peter Füreder for the help with the surveillance system and recordings. We would also like to thank the many caregivers and dogs who volunteered to participate in this study. This project was funded by the 10.13039/501100002428Austrian Science Fund (FWF: P 36853-B, P 36896-B, and W1262-B29; www.fwf.ac.at) and took place at the University of Veterinary Medicine Vienna.

## Author contributions

Conceptualization, L.H. and C.J.V.; methodology, L.H. and C.J.V.; investigation, P.v.d.W., M.M., and S.R.; scoring, P.v.d.W. and M.M.; resources, L.H.; data curation, C.J.V.; formal analysis, C.J.V.; writing – original draft, L.H. and C.J.V.; writing – review and editing, L.H., C.J.V., and S.R.; visualization, P.v.d.W., M.M., and C.J.V.; supervision, L.H., C.J.V., and S.R.; project administration, L.H.; funding acquisition, L.H.

## Declaration of interests

The authors declare no competing interests.

## STAR★Methods

### Key resources table

**Table:** 

REAGENT or RESOURCE	SOURCE	IDENTIFIER
**Deposited data**		

Raw and analysed data	This paper	DOI:https://doi.org/10.5281/zenodo.13797455

**Experimental models: Organisms/strains**		

Domestic dog	Privately owned	

**Software and algorithms**		

Loopy	Loopbio Gmbh, Vienna, Austria	http://loopb.io
R (version 4.2.2)	R Core Team	https://www.r-project.org
RStudio	RStudio Team	http://www.rstudio.com

### Experimental model and study participant details

#### Subjects

Our target sample size of 76 dogs was based on a power analysis (https://github.com/cvoelter/dog_ToM_power; Study 2; see analysis section). We recruited 108 pure-bred dogs. Based on the findings by Heberlein et al.,^49^ we preferentially recruited pure-bred dogs that they have classified as independent and family working styles or as ancient types and hunting dogs. Heberlein and co-authors found these breeds to be more likely to take their caregiver’s perspective in a stealing task. Only 74 dogs passed the pre-tests, entered the test phase and made a choice in the test phase. Five dogs were excluded during the initial exploration phase because they did not voluntarily explore the room; nine dogs were excluded in Pre-test 1 and 13 dogs in Pre-test 2 because they did not meet the pre-test criteria. Finally, seven dogs were excluded in the test phase; four of them did not approach any of the plates and three individuals were excluded due to experimenter or dog handler mistakes. One additional dog was tested but was later excluded because we were informed that the dog suffered from hearing deficiencies (this dog was assigned to the control condition and chose the seen plate first). Our final sample consisted of 73 pet dogs of various breeds including nine herding dogs, 16 retrievers, 19 terriers, 10 toy dogs, and 19 individuals from other FCI groups. Dogs were assigned pseudo-randomly to the conditions while counterbalancing age, sex, and breed as much as possible. Out of the 73 dogs, 37 dogs were assigned to the control condition (mean age ± se: 60.4 ± 6.1 months ; range: 18-146 months; 25 females, 12 males) and 36 dogs to the cue condition (mean age ± se: 61.3 ± 5.9 months ; range: 18-135 months; 23 females, 13 males).

#### Ethical statement

The experiments described in this study complied with the ARRIVE guidelines and were discussed and approved by the Ethics and Animal Welfare Committee of the University of Veterinary Medicine, Vienna (ETK-139/09/2023) in accordance with the Good Scientific Practice guidelines of the university.

Dogs in this study engaged in a non-invasive problem-solving task to obtain food at the Clever Dog Lab in Vienna, Austria.

#### Consent to participate

All owners were informed about the nature of the studies and gave written approval for their dogs to participate.

#### Consent to publish

All owners signed a consent form allowing us to publish data and media files produced as a result of their dogs’ participation in the studies.

### Method details

#### Setup

The study was conducted in a controlled environment measuring 6.05 × 3.33 m within the Clever Dog Lab at the Messerli Research Institute, University of Veterinary Medicine Vienna. The testing room was compartmentalized by an opaque floor-length curtain and a 1.2 m high fence, which featured a centrally located Plexiglas window (52 cm wide) allowing a clear view to the other side (Figure 1). Access to both sides of the partition was provided by a door located on each side. The larger section of the room served as the dog's testing compartment, while the smaller, adjacent area (the 'kitchen compartment') was furnished with a table and chair designated for the experimenter and remained inaccessible to the dog. To maintain consistency across trials, all equipment placements were marked on the floor.

Additionally, a JBL Flip 6 loudspeaker was positioned near the experimenter's workspace to deliver the two distinct auditory conditions required for the experiment. For the sound-cue condition, we utilized a Zoom H5 audio recorder to capture the sounds produced by the experimenter during the exploratory phase, specifically the sound of carrots being cut. Conversely, for the sound-control condition, we recorded ambient noise from a bustling street in Vienna, encompassing sounds from vehicles and trams. Subsequently, both audio tracks were edited to ensure uniformity in volume, frequencies, and peak levels.

Dogs' behavior was monitored using a multi-camera system comprising four units: three Panasonic HC-V777 cameras affixed to the side walls and one Axis M3045-V camera mounted on the ceiling, which provided a comprehensive 360-degree view of the room. The video and audio outputs from the cameras were transmitted via HDMI cables to a computer positioned outside the testing area. This computer was equipped with specialized recording software and a split-screen surveillance interface.

#### Procedure

The experimental procedure comprised three distinct phases: an exploration phase, a pre-test, and a final test incorporating two conditions. Each participating dog required only one visit to the Clever Dog Lab, which lasted approximately 40 minutes, including instructions provided to the caregiver and breaks between phases.

##### Exploration phase

The primary objective of the exploration phase was to familiarize the dogs with the testing environment, particularly the locations of the food plates and the positions from which they could see the experimenter’s workspace through the window between the curtains. At the beginning of the exploration, the caregiver entered the room with the dog on a leash and seated her/himself in a chair at the starting position. Concurrently, the experimenter entered the 'kitchen compartment' from the corresponding door, took a seat, and commenced cutting carrots. Upon hearing the sound of chopping, the caregiver released the dog, allowing it to explore the room and locate the plate containing three pieces of kibble (low-value reward). Ten seconds later, the caregiver called the dog back, and together they exited the room. During the exploration trials, the plate was alternately positioned at Location A and Location B. When the plate was at Location A, the dogs had the opportunity to see through the window to the carrot chopping experimenter (Figures 2A and 2B). If the dog did not direct its attention toward the experimenter—who maintained no direct eye contact—the trial was repeated until it was certain that the dog had seen the experimenter. The dogs were divided into two groups, counterbalanced for sex and experimental conditions (refer to Subjects). Half of the dogs began with the plate at Location A, while the other half commenced with the plate at Location B (Figure 2C).

##### Pre-test 1

The subsequent pre-tests aimed to assess the dogs' levels of obedience and to exclude those at the extremes of the obedience spectrum (too obedient and too disobedient). The caregiver was blindfolded in pre-tests and tests (Figure 3A). Prior to initiating the pre-tests, the caregiver communicated the known command(s) to the experimenter that instructed the dog not to take an item (e.g., "no," "nein," "stop"). After both the caregiver and dog took up the starting position within the room, the experimenter entered through the same door carrying two plates, a stopwatch, and a bag containing pieces of sausage (high-value reward). The experimenter knelt at the designated marked spot in front of the curtain, placed one piece of sausage in each plate, showed them to the dog (Figure 3B), and then positioned the plates in their respective designated areas on her left and right sides (Figure 3C). Once the plates were set, the experimenter made eye contact with the dog and issued the command not to take the sausage (e.g., "no," "nein," "stop") with a strong, low-pitched voice and a salient hand gesture (Figure 3D). In Pre-test 1, she then activated the stopwatch audibly, which signaled the blindfolded caregiver to unleash the dog. The experimenter remained focused on the stopwatch throughout the procedure.

The criterion in Pre-test 1 was reached if the dog did not approach the food plates—indicated by stepping over the diagonal lines next to them (see Figure 1)—within a period of 20 seconds. The experimenter then collected the plates from the floor while the caregiver removed the blindfold. In instances where the dog attempted to obtain the sausage by stepping over the designated diagonal line, the experimenter would promptly remove both plates, necessitating a repetition of the pre-test. If the dog continued to exhibit non-obedience for ten trials, it was excluded from further testing.

##### Pre-test 2

Upon passing Pre-test 1, Pre-test 2 was conducted to select dogs that would attempt to "steal" the sausage after the experimenter exited the room. Following the utterance of the command and accompanying hand gesture, the experimenter activated the stopwatch and left the room. The closure of the door served as the signal for the blindfolded caregiver to release the dog. The experimenter observed the dog's behavior via a surveillance screen positioned outside the testing area. After a duration of 30 seconds, the experimenter re-entered the room to retrieve the plates and allowed the caregiver to call the dog back. To pass Pre-test 2, the dogs were supposed to eat the food reward within the 30 seconds during which the experimenter was absent. This pre-test was conducted a maximum of eight times. Dogs that failed to "steal" the sausages during these trials were subsequently excluded from further participation.

##### Pre-test 1 repetition

Lastly, to ensure that the dog continued refraining from stealing the sausage in the presence of the experimenter, we repeated Pre-test 1 (same criterion as before; maximum of eight trials).

##### Test

The 'real' test commenced after a brief break during which the caregiver and the dog waited outside the test room. The experimenter arranged the two plates, each baited with one piece of sausage, at positions A and B, respectively, then exited the room. The caregiver was instructed to guide the dog into the room and assume the starting position. While outside, the experimenter played a two-minute audio track featuring either the sound of carrot chopping or ambient street noise, according to each dog's specific group assignment. Upon hearing the sound, the blindfolded caregiver released the dog. The testing concluded either when the dog finished consuming the sausages or when the two-minute playback elapsed. Consistent with previous studies, only one test trial was conducted.^39^

### Quantification and statistical analysis

#### Data scoring

We scored the following two response variables: the identity of plate (seen, unseen) the dogs approached first in the test trial and their latency to approach the first plate from the moment they were released.

#### Statistical analysis

Based on our a-priori power analysis (see below), we fitted a generalized linear model with binomial error structure and logit link function to analyze the dogs’ choice performance (binary response variable: unseen plate coded as 1, seen plate as 0). We included condition (factor with two levels: cue and control) as only test predicter with fixed effect. Additionally, we included the dogs’ sex and age (in months, z-transformed) as control predictor variables. The power simulation to determine the sample size was based on this model. For the power analysis, we had assumed a mean performance of 0.8 in the cue condition and 0.5 in the control condition; the power analysis yielded a power of 81.4% (see ESM for more details). We used a binomial test to evaluate whether the dogs’ choices deviated significantly from the expected chance value of 0.5 separately for each condition. Additionally, we fitted a model on the dogs’ first choice latency. We first tried to fit a linear mixed model, but the assumptions of normally distributed and homogeneous residuals were violated (based on visual inspection of qq-plots). We therefore fitted a GLM with gamma error structure.

Inferences with respect to the fixed effects were drawn by performing likelihood ratio tests between the full model and reduced models lacking single predictor variables. For the GLMs with gamma error structure we also checked overdispersion which was no issue (dispersion parameter: latency model: 0.87). We also checked dfbeta values but found no indication of influential cases. Finally, we also checked for collinearity, which was no issue in any of the models (maximal variance inflation factor: choice model 1.0; latency model: 1.1).

Interobserver reliability between the two coders was determined based on 26% (=19) of the subjects and was found to be excellent for both measures (latency to approach: intraclass correlation ICC1^50^: 0.995, *p* < 0.001; first approached plate: Cohen’s kappa: 1).

## Supplemental information

Document S1. Tables S1 and S2 and Method S1
