# Supplementary material for: Canine perspective taking: Anticipating the behavior of an unseen human
Source: iScience. 2025 Jan 16;28(2):111811. doi: 10.1016/j.isci.2025.111811 (PMC11821393; doi:10.1016/j.isci.2025.111811)
Supplement: Document S1. Tables S1 and S2 and Method S1 [file mmc1.pdf]

iScience, Volume *28*

Supplemental information

Canine perspective taking: Anticipating the behavior of an unseen human

Ludwig Huber, Pauline van der Wolf, Machteld Menkveld, Stefanie Riemer, and Christoph J. Völter

Methods S1: Description of a-priori power simulation, related to STAR Methods

We conducted an a-priori simulation to evaluate the power of our planned analysis (https://github.com/cvoelter/dog_ToM_power; Study 2): for the dogs' choices of the unseen over the seen bowl we assumed that 80% of dogs would choose the unseen bowl in the cue condition and 50% of the dogs in the control condition. Additionally, we assumed that the two groups (cue / control) were balanced with respect to sex. The age of the dogs was drawn randomly (between 1 and 13 years) for every simulated dataset. We combined the fixed effects into the linear predictor and generated the response variable by sampling from a binomial distribution using the inverse logit transformed linear predictor as probability of success. We simulated 1000 datasets (with 38 dogs per condition) and fitted the GLM described in the data analysis section (unseen bowl choice ~ condition + sex + age). We evaluated the models using two criteria: whether they (i) converged and (ii) whether the likelihood ratio test of condition was significant. We determined the proportion of models that fulfilled both criteria out of all simulated models. This revealed a power of 81.4%.

Table S1. Results of the binomial GLM of the choice performance (first approached bowl; confirmatory analysis)

	Estimate	*SE*	95% *CI*	$\chi 2$	*df*	*p*
(Intercept)	0.32	0.38	[-0.42, 1.08]			
Condition[1]	1.12	0.52	[0.12, 2.2]	4.85	1	0.028
Sex[2]	-0.48	0.54	[-1.54, 0.58]	0.79	1	0.375
Age[3]	-0.13	0.26	[-0.64, 0.38]	0.26	1	0.608

Notes: Reference categories: [1]*control*; [2]female; [3]age was z-transformed to a mean of 0 and a *sd* of 1.

Table S2. Results of the gamma GLM of the latency performance of the first approached bowl.

	Estimate	*SE*	95% *CI*	*Scaled deviance*	*df*	*p*
(Intercept)	0.31	0.17	[-0.02, 0.67]			
Condition[1]	0.03	0.19	[-0.35, 0.4]	0.02	1	0.89
Chosen bowl[2]	0.14	0.2	[-0.24, 0.54]	0.5	1	0.477
Sex[3]	-0.02	0.19	[-0.4, 0.37]	0.01	1	0.908
Age[4]	0.07	0.09	[-0.1, 0.25]	0.72	1	0.396

Notes: Reference categories: [1]*control*; [2]*unseen*; [3]female; [4]age was z-transformed to a mean of 0 and a *sd* of 1.
